# Combined Spinal-Epidural for Vaginal Delivery in a Parturient With Takayasu’s Arteritis

**DOI:** 10.1177/2324709616683725

**Published:** 2016-12-01

**Authors:** Sean Patrick Clifford, Paul Brian Mick, Brian Matthew Derhake

**Affiliations:** 1 University of Louisville Hospital, Louisville, KY, USA; 2 Deaconess Hospital, Evansville, IN, USA; 3 The Pain Institute, Louisville, KY, USA

**Keywords:** Takayasu’s arteritis, combined spinal-epidural, labor pain

## Abstract

Takayasu’s arteritis is a rare, progressive panendarteritis involving all layers of the arterial wall. This disease includes variable involvement of the aorta and its major branches. The most common complication with this condition is severe, uncontrolled hypertension, often leading to end organ dysfunction. We describe the management of a 27-year-old woman diagnosed with Takayasu’s arteritis that presented in labor with intense pain and underwent a combined spinal-epidural for anesthetic management. Per literature review, a combined spinal-epidural technique for planned vaginal delivery has not been described for a laboring Takayasu patient. Our technique, utilizing intrathecal opioids and a low-dose local anesthetic-opioid epidural infusion, provided adequate analgesia while maintaining hemodynamic stability throughout labor augmentation and successful vaginal delivery.

## Introduction

Takayasu’s arteritis (TA) is a rare inflammatory vascular disease most common in young, Asian women. It can cause a panarteritis with thrombosis and occlusion primarily in the aorta and its major branches, including pulmonary, and rarely coronaries arteries.^1,2^ Hypertension is seen in 72% of patients and is an important determinant of heart failure and mortality related to the disease.^3,4^ Anesthetic approaches have varied for parturients with TA, and there are reports in the literature of general anesthesia as well as neuraxial analgesia,^5-16^ including the use of a combined spinal-epidural (CSE) technique with local anesthetic alone for cesarean section.^17^ Fear of the hypotension that can be associated with neuraxial anesthesia is a significant concern in this patient population,^9^ and many of the patients described in these reports experienced hypotension requiring augmentation with intravenous medication and/or fluid boluses. We report a successful CSE with opioid-only medication injected into the intrathecal space followed by a combined local anesthetic-opioid epidural infusion. This particular technique utilized the beneficial effects of intrathecal opioid analgesia to decrease the dosage of epidural local anesthetic required to maintain the patient’s comfort. Adequate pain control with minimization of the sympathectomy produced by neuraxial local anesthetics allowed for our patient to maintain stable hemodynamics throughout her labor and delivery period.

## Case Description

A 27-year-old Asian woman (weight 68 kg, height 152 cm) with Type 3 TA^18^ diagnosed at 17 years of age presented at 39 weeks and 2 days gestation with very painful contractions and cervical dilation of 3 cm. Her obstetrical history included 3 prior miscarriages at 5, 6, and 10 weeks. Transthoracic echocardiogram from 10 days prior showed normal left ventricular systolic function with ejection fraction of 61%, left ventricular diastolic dysfunction (impaired relaxation), mild aortic regurgitation, and a right ventricle of normal size and function. Serial magnetic resonance angiogram studies showed stable multivessel disease with severe narrowing of the aorta and other major arteries. There was noted thickening of the aortic arch, severe abdominal aortic narrowing with the infrarenal aorta diameter measuring 0.7 cm, as well as bilateral carotid and brachiocephalic artery narrowing. There was no pulmonary artery involvement or renal insufficiency. No other significant past medical or surgical history was present, and medications included only prenatal vitamins. During prenatal obstetric appointments, the patient’s noninvasive blood pressure (NIBP) ranged from 112/71 mm Hg (mean arterial pressure [MAP] 85 mm Hg) to 137/101 mm Hg (MAP 113 mm Hg).

Physical exam revealed an alert, oriented, and cooperative patient with normal respiratory effort and lungs that were clear to auscultation. Cardiovascular exam yielded bilateral 1+ carotid bruits and an abdominal aortic bruit. Pulses were nonpalpable bilaterally at the femoral, pedal, posterior tibial, dorsalis pedis, brachial, and radial sites. The patient’s presenting NIBP was 137/76 mm Hg (MAP 99 mm Hg) and could only be measured from her left lower extremity. Auscultation of the heart was normal and further physical exam was unremarkable.

The consulting cardiologist suggested that peripartum hypertension would be a concern for poor outcome and recommended a cesarean section to avoid the potential of hypertension associated with pushing during a vaginal delivery. However, the patient had a strong desire to attempt vaginal delivery, and the high-risk maternal and fetal medicine obstetrician decided to allow a trial of labor with a plan for vacuum delivery to decrease the need for maternal pushing. It was agreed that conversion to cesarean section would take place immediately at any signs of maternal instability or fetal distress. Oxytocin was planned for augmentation, and neuraxial anesthesia was requested for pain control, at which time NIBP was noted to be as high as 158/88 mm Hg (MAP 109 mm Hg).

A combined spinal and epidural approach was selected with 25 µg of fentanyl injected into the intrathecal space, bringing her stated pain of 10/10 down to 0/10 within a few minutes. An epidural catheter was placed immediately after the spinal injection, and a solution of 0.1% bupivacaine and 3 µg/mL of fentanyl was started at 6 mL per hour without an initial epidural bolus. The rate was eventually increased to 8 mL/h at full cervical dilatation. As the patient became more uncomfortable during the second stage of labor, the infusion was increased to 12 mL/h to allow for adequate pain and blood pressure control. The patient delivered a healthy baby approximately 12 hours after initiation of neuraxial anesthesia with assistance of a vacuum device, as planned by the obstetrician. Apgar scores were 8 and 8 at 1 and 5 minutes, respectively. No complications were noted. The patient had an uneventful recovery period and was discharged 2 days later.

## Discussion

Several anesthetic goals were identified during the pre-anesthetic evaluation for our patient with TA. Our initial goal was to rapidly decrease the patient’s intense pain in order to limit the risk of hypertension. Her NIBP had risen to 151/91 mm Hg (MAP 111 mm Hg) prior to neuraxial anesthetic placement. Hypertension is a serious potential complication that can present in this patient population, as has been previously suggested in the literature.^5,15,18-21^ Narcotic-only intrathecal anesthesia was chosen because of the potential to rapidly decrease pain in stage 1 of labor while limiting the hypotension that often follows intrathecal local anesthetic administration.^22-24^ This coincided with the goal of maintaining adequate perfusion to both the mother and fetus by avoiding unnecessary hypotension.^25^ The patient reported an initial resolution of her pain after the intrathecal injection. A third anesthetic goal was to provide continuous pain control and hemodynamic stability throughout the course of labor and delivery. In order to achieve this goal, an epidural catheter was placed immediately after the intrathecal opiate injection to allow for continuous analgesia. Medication was infused at a basal rate without an initial bolus dose. A solution of 0.1% bupivacaine and 3 µg/mL fentanyl was selected. A fentanyl concentration of 3 µg/mL was selected instead of 2 µg/mL, the most common formulation at our institution, with the intention of improving pain control while limiting the rate of epidural infusion of local anesthetic and decreasing the risk of hypotension. The patient did not require any medication or fluid boluses to maintain her blood pressure after neuraxial analgesic administration.

The patient only pushed her button for patient controlled anesthesia on 2 occasions throughout the 12-hour labor course, once receiving a 2-mL bolus of the epidural medication, and another time receiving a 3-mL bolus. She did require 4 mg of intravenous morphine on one occasion while awaiting a change in epidural rate to take place. This patient delivered a healthy baby with no apparent complications. Afterward, she reported that her pain was very well controlled throughout the course of labor and delivery.

In conclusion, we suggest that the use of a spinal injection with opioid medication followed by a continuous epidural infusion of opioid and local anesthetic should be considered for laboring patients with TA. Several anesthetic goals were accomplished with this technique. This patient’s intense labor pain was rapidly decreased in an attempt to limit the risk of hypertension, perfusion was adequately maintained as unnecessary hypotension was avoided, and continuous pain control and hemodynamic stability were accomplished throughout the course of labor and delivery. As always, the choice of anesthesia technique should be selected based on individual patient’s presentation and comorbidities.
